# Case Report: Advanced magnetic resonance imaging findings in two cases of anaplastic papillary glioneuronal tumor: one case with glioblastoma-like progression

**DOI:** 10.3389/fonc.2025.1598058

**Published:** 2025-08-13

**Authors:** Yifan Zhang, Jialin He, Wei Xue, Jingqin Fang, Xuesong Du

**Affiliations:** ^1^ Department of Radiology, The Second Affiliated Hospital of Anhui Medical University, Hefei, China; ^2^ Department of Stomatology, The 940th Hospital of Joint Logistic Support Force of Chinese Peoples Liberation Army, Lanzhou, China; ^3^ Medical Imaging Research Center, Anhui Medical University, Hefei, China; ^4^ School of Modern Health and Regimen Industry, Anhui Sanlian University, Hefei, China; ^5^ Department of Radiology, The 940th Hospital of Joint Logistic Support Force of Chinese Peoples Liberation Army, Lanzhou, China; ^6^ Department of Ultrasound, Daping Hospital, Army Medical University, Chongqing, China

**Keywords:** papillary glioneuronal tumor, anaplastic, advanced MRI, recurrent, malignant transformation

## Abstract

Papillary glioneuronal tumors (PGNTs) are classified by the World Health Organization (WHO) as Grade I neoplasms, with only sporadic reports of anaplastic variants demonstrating aggressive clinical behavior and distinct histopathological characteristics. This study presents two cases of anaplastic PGNT, including one that ultimately progressed to glioblastoma (WHO Grade IV). The first case involved a 47-year-old female patient without a history of seizures. Magnetic resonance imaging (MRI) revealed an irregular mass containing multiple cysts and a mural nodule in the left parietal lobe. Histopathological examination confirmed the diagnosis of anaplastic PGNT. Nineteen months later, follow-up brain MRI demonstrated a recurrent mass at the prior surgical site. During the second resection, histological analysis identified glioblastoma arising from the glial component of the original tumor. The second case concerned a 7-year-old boy presenting with progressive headache. MRI showed a solid-cystic mass in the right frontal lobe accompanied by marked peritumoral edema. Postoperative pathological examination revealed anaplastic PGNT with extensive necrosis. MRI characteristics including prominent peritumoral edema, ring-enhancing cyst walls, restricted diffusion, and elevated lipid/lactate peaks may predict the aggressive nature of anaplastic PGNT. Furthermore, this case series suggests that anaplastic PGNTs harbor malignant potential to transform into more aggressive neoplasms.

## 1 Introduction

Although PGNT was first characterized as a variant of mixed glioneuronal tumor by Komori et al. (1) in 1998, it was not acknowledged as a distinct entity until 2007. In the 2016 and 2021 editions of the WHO classification of central nervous system (CNS) tumors, PGNT was classified as a Grade I neuronal and mixed neuronal-glial tumor owing to its relatively benign clinical course and benign histological features. These features include cytological benignity, absence of mitosis, vascular endothelial hyperplasia, or necrosis, along with extremely low proliferative activity (2).

However, PGNTs do not invariably manifest benign traits; they may instead display clinically aggressive tendencies or exhibit pathologically malignant attributes. The inaugural report of a PGNT with a progressively deteriorating clinical course emerged in 2006 (3). The descriptors “atypical” or “anaplastic” are utilized to delineate PGNT cases marked by aggressive biological behaviors or elevated proliferative activities. The diagnosis of anaplastic PGNT is firmly grounded in its characteristic papillary architecture, the concurrent presence of neuronal and glial elements, and a notably high proliferation index (4). Anaplastic PGNT has the potential to recur subsequent to the initial surgical excision, irrespective of whether adjuvant radiotherapy or chemotherapy is administered. Moreover, in certain cases, documented evidence has revealed tumor dissemination within the brain (5, 6), involvement of the convexity subdural space (6), and even extraneural metastasis (7).

It is worth mentioning that, prior to this study, limited documented cases have been found regarding recurrent PGNT undergoing malignant transformation, especially in terms of transitioning from a low to a high proliferation index or evolving from a low - grade to a high - grade tumor. Herein, we present two additional cases of anaplastic PGNT, one of which progressed to glioblastoma (GBM; WHO Grade IV). The present case suggests the possibility of PGNT undergoing transformation into GBM, a phenomenon with limited prior documentation.

## 2 Case description

### 2.1 Case 1

A 47-year-old woman presented in 2012 with a history devoid of seizures but complaining of headache and dizziness. Physical examination revealed anosmia and impaired fine motor function in both hands, with greater involvement of the right hand. Neurological and ophthalmologic examinations yielded no abnormal findings. Brain computed tomography (CT) demonstrated a mass with heterogeneous density in the left parietal lobe. Based on these clinical and radiological findings, the patient was initially diagnosed with glioma.

Following admission, the patient was subjected to MRI examinations, encompassing both conventional and advanced imaging sequences. The MRI features of Case 1 are detailed in **Table 1**.

**Table 1:** MRI features of the two cases.

MRI features	Case 1	Case 2
Mass Characteristics	Solid-cystic mass	Solid-cystic mass
Peritumoral	Moderate peritumoral edema	Severe peritumoral edema
T1WI	Heterogeneous hypointensity	Heterogeneous hypointensity
T2WI/FLAIR	Hyperintense signal with linear low signal intensity within the mural nodule.	Hyperintense signal with focal low signal intensity within the mural nodule.
Contrast-enhanced	Marked enhancement	Marked enhancement
DWI/ADC	Slightly restricted diffusion in the solid component, and reduced ADC	Slightly restricted diffusion in the solid component, and reduced ADC
DTI	Disruption of white matter tracts	/
SWI	Linear or focal hypointensities within both the solid component and the cyst wall	/
¹H-MRS	Increased Cho and NAA levels in solid component. Elevated Lip/Lac peak, while decreased or undetectable Cho and NAA levels in cystic component	Increased Cho and NAA levels in solid component
DSC-MRI	Elevated rCBV and rCBF in solid component	Elevated rCBV and rCBF in solid component

The left parietal mass, with dimensions of 5.3 × 4.4 × 4.0 cm, presented an irregular contour, characterized by the presence of multiple cysts and a mural nodule. It was situated adjacent to the left lateral ventricle, extending into both the cortical and subcortical regions. Moderate peritumoral edema was evident, leading to the displacement of adjacent brain tissue and a rightward shift of the midline structures.

On T1-weighted imaging (T1WI), both the cystic components and the mural nodule exhibited heterogeneous hypointensity, whereas they appeared hyperintense on T2-weighted imaging (T2WI) and fluid-attenuated inversion recovery (FLAIR) sequences. Within the mural nodule on T2WI, a linear low-signal intensity was discernible. Postcontrast scans revealed prominent enhancement of the solid nodule and the cyst wall.

**Figure 1:**
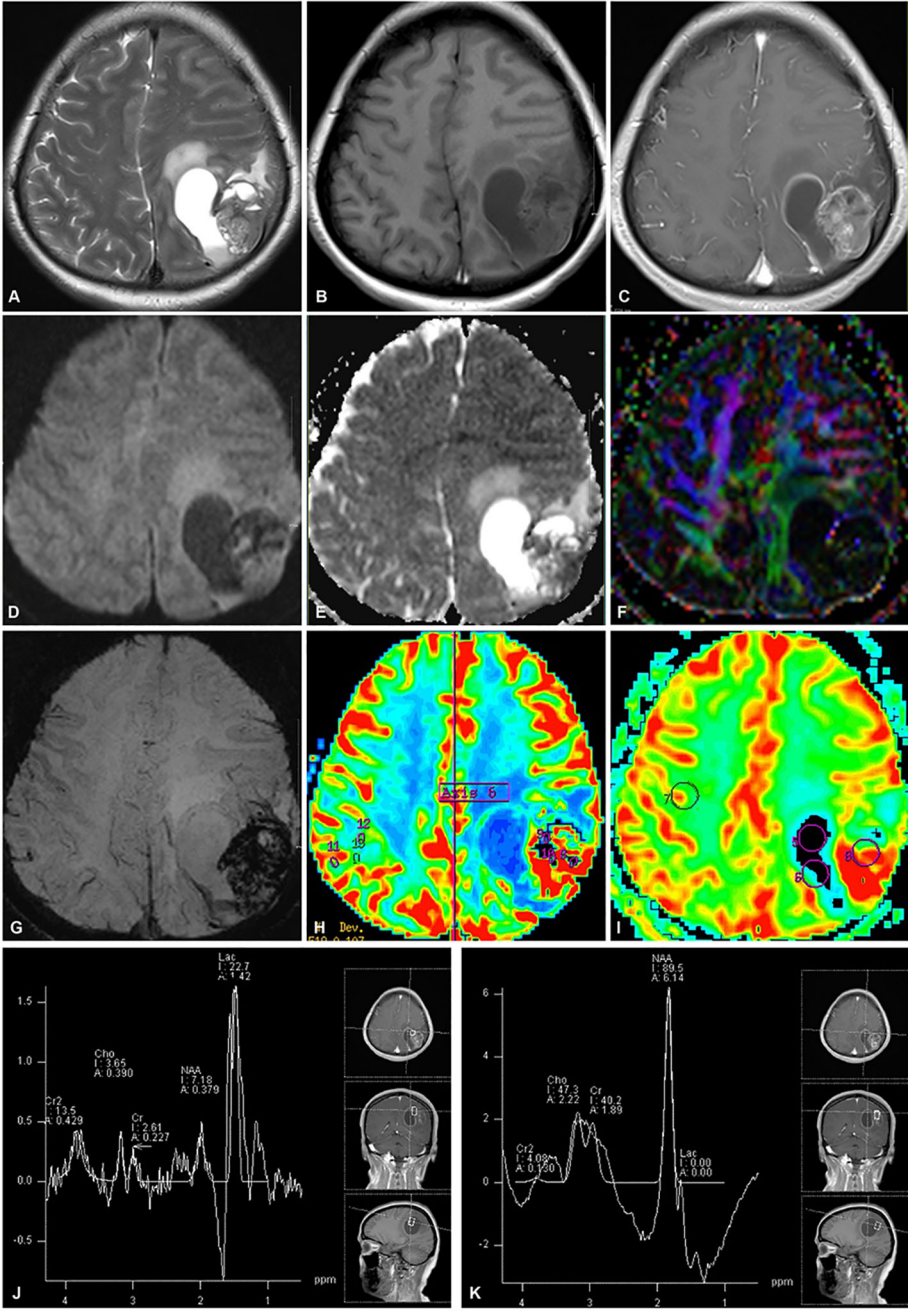
Preoperative conventional and advanced MRI (Case 1). **(A, B)** Axial T2WI **(A)** and T1WI **(B)** reveal a left parietal lobe mass with heterogeneous intensity and minimal edema, resulting in a midline shift. **(C)** With contrast medium, the tumor solid component and cyst wall exhibit a heterogeneous and ring-enhancing pattern, respectively. **(D, E)** The solid part demonstrates slight restricted diffusion **(D)** and a low ADC **(E)**. **(F)** DTI demonstrates disruption of white matter fibers within the tumor region. **(G)** SWI reveals hemorrhage within the tumor. **(H, I)** DSC-MRI exhibits a solid part with high rCBV **(H)** and rCBF **(I)**. **(J, K)** High lipid/lactate (Lac/Lip) (1.33 ppm) in the cystic portion **(J)** and elevated Cho (3.2 ppm) and NAA (2.0 ppm) in the solid part **(K)** are observed on ^1^H-MRS.

Diffusion-weighted imaging (DWI) indicated slightly restricted diffusion within the solid component, corresponding to a decreased apparent diffusion coefficient (ADC). Diffusion tensor imaging (DTI) demonstrated a disruption of white matter tracts within the tumor area, with adjacent tracts showing displacement or swelling. On susceptibility-weighted imaging (SWI), in addition to the hypointensity in the region corresponding to the T2WI low signal, linear or focal hypointensities were identified within both the solid component and the cyst wall. Metabolic profiling of the tumor was conducted using proton magnetic resonance spectroscopy (¹H-MRS) with the following parameters: repetition time/echo time (TR/TE) = 1700/135 ms. ¹H-MRS revealed significant metabolic disparities between the cystic and solid portions. The cystic component displayed a markedly elevated lipid/lactate (Lip/Lac) peak at 1.33 ppm, while the levels of choline (Cho, 3.2 ppm) and N-acetylaspartate (NAA, 2.0 ppm) were substantially reduced or undetectable. In contrast, the solid component showed increased Cho and NAA levels, especially in regions with homogeneous signal intensity. Dynamic susceptibility contrast (DSC)-MRI demonstrated elevated relative cerebral blood volume (rCBV) and relative cerebral blood flow (rCBF) in both the solid component and the cyst wall (**Figure 1**).

In February 2012, a left parietal craniotomy accompanied by gross - total resection (GTR) was carried out. Histopathological examination of the surgical specimen provided definitive confirmation of the diagnosis: anaplastic papillary glioneuronal tumor (classified as WHO Grade III). However, the patient and her family opted to forgo postoperative adjuvant chemoradiotherapy. She was subsequently discharged on the 13th postoperative day (POD13).

**Figure 2:**
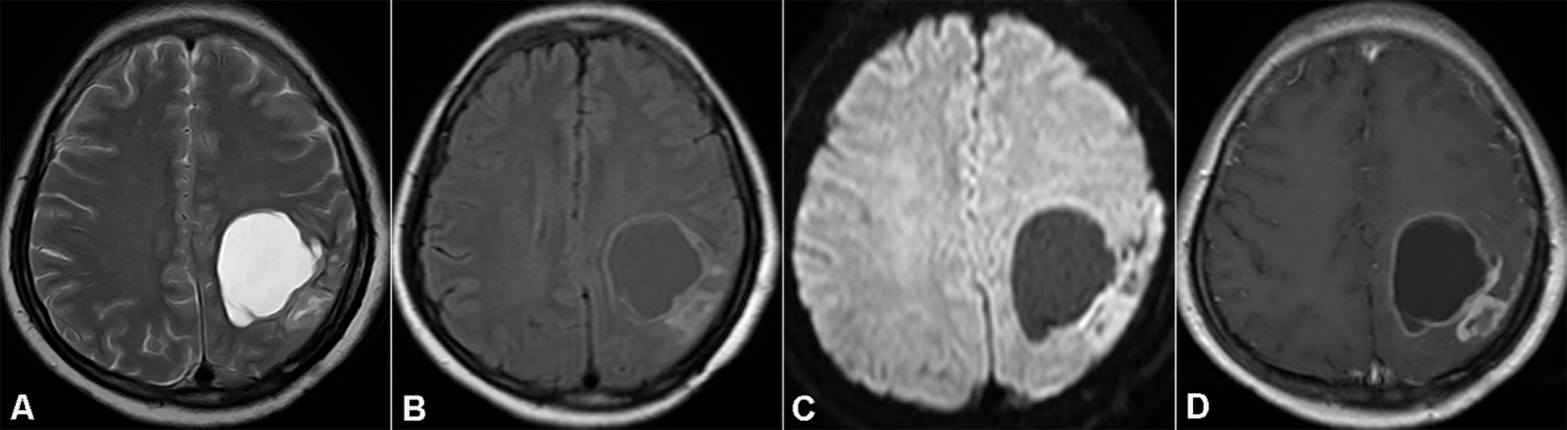
Magnetic resonance imaging (MRI) showing recurrent tumor in the initial resection cavity (Case 1). **(A, B)** Axial T2WI and FLAIR. **(C)** DWI demonstrates notable diffusion restriction in the solid part of the recurrent tumor. **(D)** Axial T1WI with contrast medium shows enhancement of the mural nodule and cyst wall.

Nineteen months after the initial surgery, specifically in September 2013, the patient was readmitted to the hospital. She presented with a one - month history of persistent and progressively worsening headaches, along with newly developed limb paresthesia. A brain MRI scan revealed a cystic mural nodule at the site of the previous surgical resection. DWI demonstrated significantly restricted diffusion within the solid component of the lesion. Contrast enhancement was evident not only in the cyst wall and mural nodule but also in the adjacent thickened pia mater (as shown in **Figure 2**).

The patient then underwent a second craniotomy, during which a GTR of the recurrent lesion was achieved, including the enhancing cyst wall. Histopathological examination confirmed that the recurrent tumor was glioblastoma multiforme (GBM), which had arisen from the malignant transformation of the glial component of the primary neoplasm. Despite the medical team’s recommendation, the patient declined adjuvant chemotherapy. She remained free of disease for approximately nine months following the second surgery. However, a follow - up MRI conducted in July 2014 detected recurrent GBM within the cavity of the previous resection.

### 2.2 Case 2

A 7-year-old boy presented in July 2017 with a 2-month history of progressive, holocranial headache accompanied by vomiting and postural dizziness. Neurological examination revealed mild left-sided hemiparesis (Medical Research Council grade 4/5) with hyporeflexia in the biceps and patellar tendons. Cranial nerve assessment and right-sided motor-sensory functions remained intact. Initial non-contrast brain CT demonstrated a hyperdense, space-occupying lesion in the right cerebral hemisphere with subtle perilesional hypodensity.

**Figure 3:**
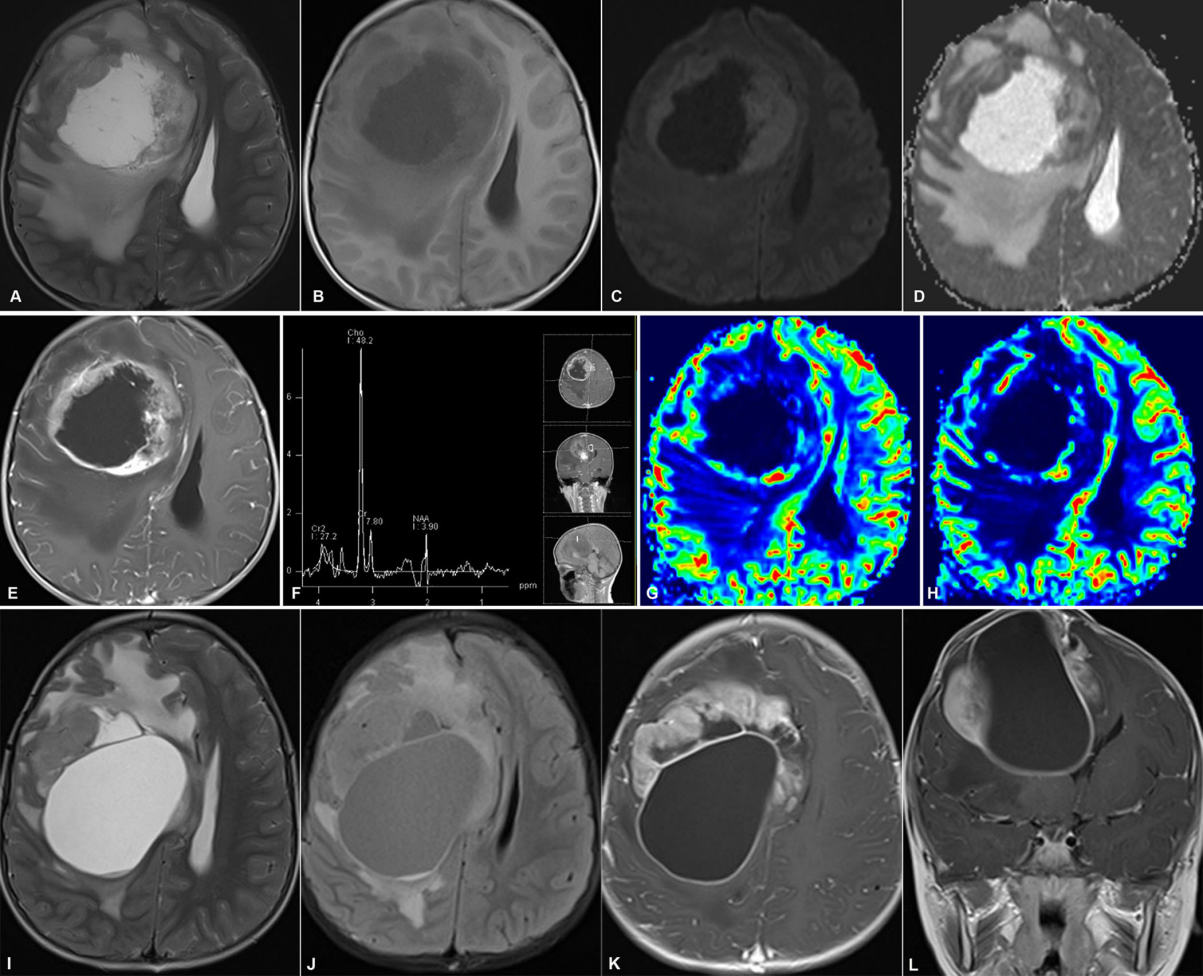
Representative MRI of primary and recurrent tumor (Case 2). **(A, B)** Axial T2WI and T1WI show a solid-cystic tumor in the right frontal lobe, causing substantial peritumoral edema and a midline shift. **(C, D)** The solid part of the tumor shows marked restricted diffusion on DWI **(C)** and a decreased ADC value **(D)**. **(E)** The solid part and the cyst wall exhibit notable enhancement. **(F)** MRS displays high Cho and a low NAA value in the solid area. **(G, H)** DSC-MRI reveals focal elevations in rCBV and rCBF. **(I–L)** Follow-up MRI demonstrates tumor regrowth in the previous surgical region. **(I, J)** Axial T2WI and FLAIR. **(K, L)** Axial and coronal T1WI with contrast medium. The recurrent tumor, with a larger cystic part and multiple nodules, demonstrates notably heterogeneous enhancement.

A brain MRI scan conducted at our hospital unveiled a solid - cystic mass situated in the right frontal lobe. The MRI features of Case 2 are detailed in **Table 1**. The mass was well - defined, with a surrounding rim of marked edema that induced a leftward displacement of the midline structures. The lesion, measuring 6.7 × 7.0 × 7.7 cm, displayed heterogeneous hypointensity on T1WI and hyperintensity on T2WI. On DWI, the solid component of the mass exhibited significantly restricted diffusion, accompanied by a reduction in the ADC. After the administration of contrast agent, the solid part and the cyst wall demonstrated prominent enhancement. MRS revealed a marked elevation in Cho levels and a decrease in NAA levels within the solid portion. On DSC - MRI, the solid part of the mass showed higher rCBV and rCBF values compared to those of the contralateral normal brain parenchyma (as depicted in **Figures 3A–H**).

Based on these findings, high - grade gliomas and anaplastic ependymomas were considered as potential diagnoses. Subsequently, the patient underwent a right frontotemporal craniotomy along with tumor resection. A postoperative brain CT scan confirmed GTR of the tumor. Histopathological examination identified the tumor as anaplastic PGNT with extensive necrotic regions. Due to economic constraints, the patient did not receive adjuvant therapy. Upon discharge in August 2017, his clinical symptoms, including headaches, were significantly alleviated. Approximately five months later, in December 2017, the patient had a brain MRI in the outpatient department. The MRI results indicated tumor regrowth, presenting as a cystic mass with multiple nodules within the previous surgical cavity (as shown in **Figures 3I–L**). The patient refused a second surgical intervention and ultimately succumbed to the progression of the recurrent tumor in April 2018.

Detailed information on imaging equipment, coil/setup, scanning protocols, workstation specifications, and post-processing methods for both conventional and advanced MRI has been provided in the **Supplementary Material**.

## 3 Pathological findings

**Figure 4:**
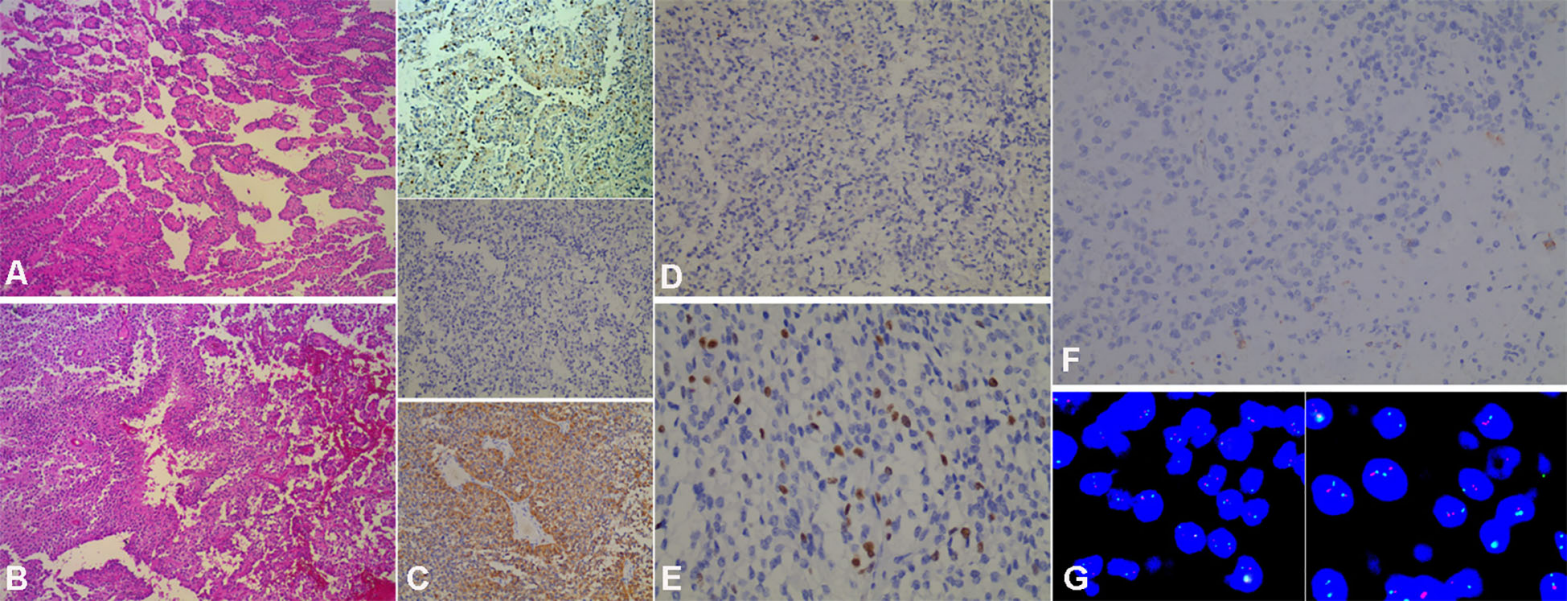
Histopathological findings of anaplastic PGNT. **(A, B)** Representative HE staining exhibits the tumor’s pseudopapillary structure **(A)** and necrosis **(B)**. **(C)** The initial tumor of Case 1 shows NeuN-positive cells within the papillary architecture, representing the neuronal component (top). NeuN staining is negative in the recurrent tumor (middle). Strong cytoplasmic positivity for GFAP in the recurrent tumor reveals the glial origin of the tumor cells (bottom). **(D)** Immunohistochemical staining for IDH-1 in the recurrent tumor reveals no IDH-1 mutation. **(E)** The Ki-67 index is up to 25% in the recurrent tumor. **(F)** IDH-1 staining reveals a wild-type IDH-1 in the tumor of Case 2. **(G)** Fluorescence *in situ* hybridization determines that the deletion rate of 1p is 22% (left) and that of 19q is 23% (right). [**(A, B, C)**-top: ×100; **(C)**-middle/bottom, (**D, F**): ×200; (**E, G)**: ×400].

Histopathological examination unveiled several common features shared by the two cases. Notably, a pseudopapillary architecture was observed, which was characterized by hyalinized blood vessels lined with glial components and interspersed neuronally - differentiated cells within the pseudopapillary framework (as depicted in **Figure 4A**). In addition, the initial histopathological evaluation of Case 1 revealed hemorrhagic foci, palisading necrosis, and microvascular proliferation (shown in **Figure 4B**). When compared to the primary specimen, the recurrent tumor in Case 1 demonstrated a reduction in hemorrhage, vascular proliferation, palisading necrosis, and papillary structures. Moreover, Case 2 also presented with extensive necrosis, while calcification was not identified in either of the two cases.

Immunohistochemically, the primary tumor in Case 1 exhibited positive staining for neuronal nuclei (NeuN), as illustrated in **Figure 4C**, accompanied by a Ki - 67 labeling index of 10%. In the recurrent tumor, there was prominent glial fibrillary acidic protein (GFAP) immunoreactivity, along with a loss of NeuN expression, also shown in **Figure 4C**. No mutations in isocitrate dehydrogenase 1 (IDH - 1) were identified (**Figure 4D**), while oligodendrocyte transcription factor 2 (Olig - 2) maintained positive staining. The Ki - 67 labeling index escalated to 25% in the recurrent lesion, as depicted in **Figure 4E**. For Case 2, immunohistochemical staining demonstrated positive results for both GFAP and synaptophysin (Syn), with no signs of IDH - 1 mutations, as presented in **Figure 4F**. Fluorescence *in situ* hybridization (FISH) analysis revealed that 22% of cells exhibited 1p deletion and 23% showed 1q deletion, as seen in **Figure 4G**. Based on institutional criteria (where a deletion is determined when the proportion is ≥30%), neither 1p nor 19q loss was confirmed. The Ki - 67 labeling index for Case 2 was 25%.

## 4 Discussion

PGNT represents a rare CNS neoplasm, categorized within the group of neuronal and mixed neuronal - glial tumors as per the 2021 WHO Classification of CNS Tumors. Histologically, it exhibits papillary configurations with central fibrovascular cores, accompanied immunohistochemically by concurrent expression of glial markers (GFAP) and neuronal markers (NeuN, Syn) (8). These defining features were consistently observed in both the initial surgical specimen from Case 1 and the resected tissue from Case 2. Molecular profiling revealed distinct classifications: In Case 1, isocitrate dehydrogenase-1 (IDH-1) mutation analysis performed on the surgical sample of the recurrent tumor confirmed that the case belonged to the isocitrate dehydrogenase wild-type glioblastoma (GBM) molecular subtype. In Case 2, FISH failed to detect 1p/19q codeletion, thereby excluding oligodendroglioma from the differential diagnosis.

Clinically, PGNT primarily impacts young adults, with the mean age at diagnosis being 26.9 years and a slight male preponderance (1.42:1). Over 80% of patients are under the age of 40 (9). The clinical manifestations are non - specific and often mimic those of other CNS lesions, encompassing seizures, headaches, or symptoms of intracranial hypertension. In this particular series, headache emerged as the most common symptom, in line with previous research findings. Anatomically, PGNT exhibits a strong tendency to occur in supratentorial regions, especially the frontal and temporal lobes, frequently involving the periventricular area (10). Less frequently encountered sites include the cerebellar hemisphere, cerebellar vermis (11), fourth ventricle (12), lateral ventricle (13, 14), third ventricle (15), and pineal gland (16, 17). Although the majority of reported cases manifest an indolent clinical course and demonstrate favorable responses to treatment (18), atypical or anaplastic variants have surfaced since 2006. Notably, the current Case 1 exemplifies malignant transformation to GBM. This variability highlights the existing gaps in our comprehension of the full biological spectrum of PGNT.

PGNT continues to be inadequately characterized in radiological terms owing to its rarity and the relatively small proportion of anaplastic cases reported thus far. Consequently, there is a pressing need for further clarification regarding the MRI features that can differentiate typical PGNT from its anaplastic counterpart.

In conventional MRI: PGNT manifests four main morphological patterns on MRI: cysts with mural nodules (the most prevalent), cystic - solid masses, purely cystic lesions, and purely solid tumors (9, 18). The current Case 1 serves as an illustrative example of the cyst - with - mural - nodule variant. Under standard MRI protocols, the cystic component of PGNT typically displays a cerebrospinal fluid (CSF) - like signal intensity. In contrast, the solid mural nodule exhibits hypointensity on T1WI and hyperintensity on T2WI. The patterns of contrast enhancement are variable: the solid component or mural nodule shows heterogeneous enhancement, while the cyst wall may either remain non - enhancing or demonstrate ring enhancement. Emerging evidence indicates that cyst wall enhancement is associated with increased tumor proliferative activity (19), a finding corroborated by the ring - enhancing cyst wall observed in Case 1.

The extent of peritumoral edema around PGNT varies, with moderate - to - severe edema being more frequently linked to anaplastic variants or larger tumor volumes (6). In this series, Case 1 presented with moderate peritumoral edema, and Case 2 exhibited marked peritumoral edema. Although calcifications are occasionally reported in PGNT, no calcified foci were detected in the current cases.

In advanced MRI: Previous research has indicated that there are no notable differences in DWI characteristics between typical and anaplastic PGNT. Both variants typically exhibit a mixture of iso - and hypointensity or pure hypointensity on DWI, which implies the absence of diffusion restriction (4). Yadav et al. conducted an analysis of seven cases of Grade I PGNT and found that four out of five assessable cases showed no diffusion restriction on DWI, while only one recurrent tumor displayed patchy diffusion restriction within its solid components (21). Similarly, Wang et al. documented a case of anaplastic PGNT without any detectable diffusion restriction (4). In stark contrast, the current Case 1 demonstrated mild and marked diffusion restriction in the solid components of the primary and recurrent tumors, respectively. Meanwhile, Case 2 exhibited pronounced diffusion restriction in the solid portion. The elevated proliferative index (Ki - 67: 25%) in the recurrent tumor of Case 1 and in the lesion of Case 2 may well explain these discrepancies in DWI findings compared to previous reports.

MRS revealed increased Cho levels in the solid components of both cases, which is in line with the findings of Wang et al. and Yadav et al. (4, 21). Notably, Case 1 showed elevated NAA in its solid component, potentially indicating a significant presence of neuronal or neuronally differentiated cells. Additionally, Case 1 demonstrated markedly increased Lip/Lac peaks in the cystic portion on MRS, suggesting intratumoral necrosis and possible malignant transformation. This is because elevated Lip/Lac ratios are commonly observed in high - grade malignancies.

DSC - MRI showed elevated rCBV values in the solid components of both cases, which is indicative of extensive neovascularization. This finding is consistent with previous reports (21, 22) and supports the anaplastic nature of our cases. Given that markedly increased rCBV is closely associated with malignant behavior and a poor prognosis in gliomas, it further reinforces the significance of our findings.

Intratumoral hemorrhage in PGNT is a rare yet clinically significant finding, potentially resulting from thin - walled vessels within the solid tumor components or abnormally hyalinized vessels in the papillary regions. Radiologically, such hemorrhagic cases may mimic high - grade carcinomas (20). SWI can offer valuable insights into the microvascular or hemorrhagic characteristics of tumors (23). In Case 1, SWI revealed intratumoral hemorrhage, which was confirmed by postoperative histopathology to originate from fragile vessels in the solid nodule. This suggests that intratumoral hemorrhage may serve as an indicator of the potential for malignant transformation and can be effectively monitored using SWI. However, the SWI shows a substantial amount of intratumoral hemorrhage in Case1, which may affect the analysis of DSC-MRI. Therefore, it is necessary to collect more anaplastic PGNT cases in subsequent studies to validate the relationship between relative cerebral blood volume (rCBV) and its malignant behavior.

Currently, the therapeutic modalities available for PGNT encompass GTR, subtotal resection, adjuvant radiotherapy, chemotherapy, or a multimodal treatment approach. Although treatment protocols for PGNT cases with elevated Ki - 67 indices exhibit considerable heterogeneity, GTR remains the linchpin for enhancing survival outcomes (19). A systematic analysis of 132 reported cases conducted by Ahmed AK et al. demonstrated that GTR alone yielded superior 2 - year progression - free survival (PFS) rates compared to subtotal resection alone (p < 0.05). Notably, the Ki - 67 labeling index serves as a more potent negative prognosticator for PFS than the extent of surgery, with a high Ki - 67 (>5%) predicting significantly reduced 5 - year PFS rates (9). Consequently, PGNTs with high proliferative activity are more likely to be treated with adjuvant radiotherapy compared to those with low proliferative activity. In our patient cohort, all patients underwent GTR without receiving adjuvant radiochemotherapy. This treatment strategy may account for the early tumor recurrence observed in Case 1 (19 months postoperatively) and Case 2 (5 months postoperatively). These findings highlight the imperative need for post - surgical adjuvant therapy in PGNT with high Ki - 67 indices, especially when complete anatomical resection is unattainable. Future prospective studies should assess the optimal sequencing of radiotherapy and chemotherapy in this patient population.

The isocitrate dehydrogenase gene (IDH) encodes isocitrate dehydrogenase, one of the crucial enzyme families involved in the tricarboxylic acid cycle. Currently, two subtypes of IDH genes associated with gliomas, IDH1 and IDH2, are primarily observed in IDH-mutant astrocytomas and IDH-mutant oligodendrogliomas with 1p/19q codeletion (8). IDH-mutant gliomas exhibit higher sensitivity to radiotherapy and chemotherapy (such as temozolomide) (24). Furthermore, targeted drugs against IDH mutations (Vorasidenib) have demonstrated significant efficacy (25), with markedly better prognosis compared to patients with IDH-wildtype gliomas. 1p/19q codeletion results from an unbalanced translocation between the long arm of chromosome 1 (1q) and the short arm of chromosome 19 (19p), leading to the formation of a fusion chromosome 1q/19p, this process causes the complete loss of one copy of the short arm of chromosome 1 (1p) and one copy of the long arm of chromosome 19 (19q), while the other copy of chromosome 1 and chromosome 19 remain intact (26). Incomplete or partial deletions of the 1p/19q chromosomal arms do not meet the diagnostic criteria for oligodendroglioma but can occur in cases of IDH-wildtype glioblastoma. Oligodendrogliomas harboring both 1p/19q codeletion and IDH mutations exhibit slower growth rates and demonstrate enhanced sensitivity to combination chemotherapy with procarbazine + lomustine + vincristine (PCV) and temozolomide monotherapy, resulting in significantly prolonged overall survival (8).

Although PGNT generally manifest an indolent clinical progression and exhibit benign histopathological characteristics, a certain subset of these tumors displays aggressive biological behavior. Case 1 in the present study offers compelling evidence of the malignant transformation of PGNT into high - grade neoplasms. Several advanced MRI biomarkers have the potential to predict aggressive phenotypes in anaplastic PGNT. These include moderate - to - severe peritumoral edema, a significant mass effect accompanied by midline shift, cyst wall ring enhancement on contrast - enhanced sequences, intratumoral hemorrhage on SWI, restricted diffusion on DWI, and an elevated Lip/Lac peak ratio on MRS. Future multicenter studies involving larger patient cohorts are essential to validate these imaging predictors and establish standardized MRI - based grading criteria for PGNT. Such endeavors would enable the formulation of personalized treatment strategies and enhance prognostic stratification for this rare tumor entity.

## Data Availability

The datasets presented in this article are not readily available because of ethical and privacy restrictions. Requests to access the datasets should be directed to the corresponding author/s.

## Glossary

PGNT: Papillary glioneuronal tumors
MRI: Magnetic resonance imaging
WHO: World Health organization
CNS: Central nervous system
GBM: Glioblastoma
CT: Computed tomography
MRI: Magnetic resonance imaging
DWI: Diffusion-weighted imaging
¹H-MRS: ¹H-magnetic resonance spectroscopy
PWI: Perfusion-weighted imaging
rCBV: relative cerebral blood volume
GTR: Gross total resection
Cho: Choline
NAA: N-Acetylaspartate
NeuN: Neuronal nuclei
GFAP: Glial fibrillary acidic protein
Olig-2: Oligodendrocyte transcription factor 2
Syn: Synaptophysin
IDH-1: Isocitrate dehydrogenase 1.
