# Supplementary material for: Case Report: Advanced magnetic resonance imaging findings in two cases of anaplastic papillary glioneuronal tumor: one case with glioblastoma-like progression
Source: Front Oncol. 2025 Aug 13;15:1598058. doi: 10.3389/fonc.2025.1598058 (PMC12381771; doi:10.3389/fonc.2025.1598058)
Supplement: Supplementary file 1 [file DataSheet1.docx]

**Magnetic resonance scanning**

MRI scans were performed on a 3.0 Tesla MRI scanner (Magnetom Verio, Siemens Medical Solutions, Erlangen, Germany) with an 16-channel head coil. Sequence parameters were as follows:

T1WI, TR/TE=250/2.67ms, SL=5mm, FOV=230x230mm;

T2WI, TR/TE=4900/96ms, SL=5mm, FOV=230x230mm;

FLAIR, TR/TE=8000/94ms, SL=5mm, FOV=230x230mm;

DWI-MRI: TR/TE=6600/100ms, SL=5mm, b=0, 500, 1,000sec/mm^2^;

DTI-MRI: TR/TE=3500/80ms, SL=5mm, b=0, 1000sec/mm^2^, the scanning range extends from the vertex of the skull to the plane of the foramen magnum, with diffusion weighting collected across 30 directions;

SWI-MRI: TR/TE=37/20ms, SL=1.5mm, FA=15°;

¹H-MRS: MRS employs single-voxel imaging using Point-Resolved Spectroscopy (PRESS), TR/TE=1700/135ms, FOV=160x160mm, NEX 3;

DSC-MRI: The spin-echo echo-planar imaging parameters were as follows: TR=1500ms, SL=4mm, TE=30ms, FOV=230x230mm, matrix=128x128, FA=90°, and NEX=1.0. During 90 consecutive scans, 0.1 mmol/kg gadopentetate was injected through the basilic vein or median cubital vein at 4 mL/s at the 9^th^ phase.

**Data processing:**

A Siemens syngo MR Workstation, and syngoMMWP software (version: VE36A) was used to analyze the DSC-MRI data. A compartment model was selected as the hemodynamic model, the arterial input function (AIF) was calculated using the middle cerebral artery, and the time and signal intensity curve of the brain were obtained by AIF. Then, the software calculated CBV and CBF map.

DWI maps, ADC maps, SWI maps, white matter fiber tract images, metabolite distribution maps, metabolite ratio maps, and overlay maps combining metabolic and anatomical images were calculated automatically by the Siemens syngo MR Workstation.
